# Rapid Identification of the SNP Mutation in the *ABCD4* Gene and Its Association with Multi-Vertebrae Phenotypes in Ujimqin Sheep Using TaqMan-MGB Technology

**DOI:** 10.3390/ani15152284

**Published:** 2025-08-05

**Authors:** Yue Zhang, Min Zhang, Hong Su, Jun Liu, Feifei Zhao, Yifan Zhao, Xiunan Li, Yanyan Yang, Guifang Cao, Yong Zhang

**Affiliations:** 1 College of Veterinary Medicine, Northwest A&F University, Yangling 712100, China; 18004809342@163.com (Y.Z.); tom4ask@sohu.com (J.L.); 2 College of Veterinary Medicine, Inner Mongolia Agricultural University, Hohhot 010018, China; zhangmin5400@126.com (M.Z.); hongsu1995@126.com (H.S.); imauzff@126.com (F.Z.); yifanz311@163.com (Y.Z.); lixiunan2368@163.com (X.L.); 3 Animal Embryo and Developmental Engineering Key Laboratory of Higher Education, Institutions of Inner Mongolia Autonomous Region, Hohhot 010018, China; 4 Inner Mongolia Academy of Agricultural and Animal Husbandry Sciences, Hohhot 010000, China; swallow_0088@163.com; 5 School of Life Sciences, Inner Mongolia University, Hohhot 010021, China

**Keywords:** Ujimqin sheep, multi-vertebrae, *ABCD4* gene, single nucleotide polymorphism, TaqMan-MGB detection, molecular marker

## Abstract

Ujimqin sheep, known for its distinctive multi-vertebrae phenotypes (T13L7, T14L6, and T14L7) and economic value, has garnered significant attention. However, conventional phenotypic detection methods suffer from low efficiency and high costs. In this study, based on a key SNP locus (*ABCD4* gene, Chr7:89393414, C > T) identified through a genome-wide association study (GWAS), a TaqMan-MGB (minor groove binder) genotyping system was developed. the objective was to establish a high-throughput and efficient molecular marker-assisted selection (MAS) tool. Specific primers and dual fluorescent probes were designed to optimize the reaction system. Standard plasmids were adopted to validate genotyping accuracy. A total of 152 Ujimqin sheep were subjected to TaqMan-MGB genotyping, digital radiography (DR) imaging, and Sanger sequencing. the results showed complete concordance between TaqMan-MGB and Sanger sequencing, with an overall agreement rate of 83.6% with DR imaging. For individuals with T/T genotypes (127/139), the detection accuracy reached 91.4%. This method demonstrated high specificity, simplicity, and cost-efficiency, significantly reducing the time and financial burden associated with traditional imaging-based approaches. the findings indicate that the TaqMan-MGB technique can accurately identify the T/T genotype at the SNP site and its strong association with the multi-vertebrae phenotypes, offering an effective and reliable tool for molecular breeding of Ujimqin sheep.

## Simple Summary

This study developed a fast, cost-effective genetic test to identify Ujimqin sheep with extra vertebrae, a trait linked to higher meat yield. Researchers targeted a specific gene change (SNP: *ABCD4*, Chr7:89393414 C > T) known to cause extra vertebrae. We designed a TaqMan-MGB test using fluorescent probes to detect this SNP in blood DNA. Validated on 152 sheep, the test showed 100% agreement with gold-standard DNA sequencing (Sanger method). Compared to DR imaging (83.6% overall agreement), it accurately detected 91.4% of multi-vertebrae sheep (127/139 animals). The test is simple, high-throughput, and significantly cheaper than DR imaging. This tool enables early, efficient selection of breeding sheep with the desirable multi-vertebrae trait, accelerating genetic improvement programs for Ujimqin sheep farmers.

## 1. Introduction

Ujimqin sheep, known for their unique multi-vertebrae phenotypes (T13L7, T14L6, and T14L7 types) and high-quality meat, hold considerable economic value in the Xilin Gol League of Inner Mongolia. Multi-vertebrae sheep have been shown to possess an increased carcass weight of 1.2–3.5 kg compared to normal individuals (T13L6 type), and this trait is stably heritable through selective breeding [1].

In recent years, there has been growing research interest in the production traits of livestock animals, particularly in large animal species such as cattle, sheep, and pigs. Genome-wide association studies (GWAS) primarily advances genetic improvement of economically vital traits, including reproductive performance, disease resistance, growth efficiency, carcass quality, and longevity indicators [2,3,4]. Previous research from our laboratory identified a significant association between the ABCD4 gene and the multi-vertebrae trait in Ujimqin sheep [5]. Furthermore, Han et al. demonstrated that ABCD4 is a strong candidate gene closely linked to growth traits in Zhongwei goats [6]. Additionally, Niu et al. reported that ABCD4 influences vertebral number in Beijing Black pigs [7]. Consequently, the ABCD4 gene emerges as a promising candidate for further experimental investigation.

GWAS have revealed that an SNP located in exon 18 of the *ABCD4* gene on chromosome 7 (Chr7:89393414, C > T) is significantly associated with multi-vertebrae traits (*p* < 10^−6^) [7]. This mutation results in an amino acid substitution from arginine (Arg) to glutamine (Gln), which may alter protein conformation and affect vitamin B12 transport, disrupting the synchronization of the somitogenesis clock and leading to a variation in the number of thoracolumbar vertebrae [8,9,10]. However, current phenotypic detection methods have notable limitations. In addition to requiring specialized equipment and anatomical expertise, digital radiography (DR) imaging is prone to misidentification at the thoracolumbar junction and incurs high costs. By comparison, Sanger sequencing offers precise results. However, it involves a laborious process that is unsuitable for large-scale screening [11]. Therefore, the development of efficient, high-throughput SNP genotyping technologies is essential for molecular breeding applications. TaqMan-MGB technology combines specific probes and dual primer design with real-time fluorescent signal detection, offering high sensitivity and specificity [12,13,14]. Its simplicity and cost-effectiveness make it a practical solution for use in primary breeding facilities.

This study developed a TaqMan-MGB genotyping system based on the GWAS-identified SNP site (Chr7:89393414, C > T) and optimized the assay using standard plasmids. Its accuracy was validated by comparing genotyping results with DR imaging and Sanger sequencing data from 152 Ujimqin sheep. Furthermore, the correlation between the SNP mutation (T/T genotype) and the multi-vertebrae phenotypes was evaluated. This study provides an efficient tool for marker-assisted selection in Ujimqin sheep breeding programs.

## 2. Materials and Methods

### 2.1. Ethical Statement

All experimental procedures in this study were approved and authorized by the Animal Care and Use Committee of Inner Mongolia Agricultural University, Inner Mongolia Autonomous Region, China (Permit No. 2020008).

### 2.2. Experimental Materials

All Ujimqin sheep used in this study were sourced from the Ujimqin Stud Farm in Xilin Gol League, Inner Mongolia Autonomous Region. A total of 152 two-year-old adult rams were selected for the experiment, and blood samples and DR imaging data were collected. For each individual, 2–3 mL of venous blood was drawn into EDTA-containing collection tubes, temporarily stored at 4 °C, and subsequently preserved at −20 °C for long-term storage. DR imaging was performed to obtain radiographic images of the vertebral column.

### 2.3. Experimental Methods

#### 2.3.1. Genomic DNA Extraction

Genomic DNA was extracted from whole blood samples using a magnetic bead-based nucleic acid extraction kit (Ex-DNA Whole Blood Genomic Kit 3.0, Tianlong, China, Cat# T146). DNA concentration and purity were assessed using a NanoDrop™ 2000 UV-visible spectrophotometer (Thermo Fisher Scientific, Waltham, MA, USA).

#### 2.3.2. DR Imaging for Vertebral Phenotype

To determine the number of vertebrae in Ujimqin sheep, a portable DR system (MIKASA8015, Hiroshima, Japan) was used to acquire spinal X-ray images from 152 individuals. Images were digitally stitched to reconstruct the full vertebral column and identify the vertebrae count.

#### 2.3.3. Design of TaqMan-MGB Probes and Primers

Based on the identified SNP site (Chr7:89393414, C > T) located in exon 18 of the *ABCD4* gene and its potential association with the multi-vertebrae phenotypes [1], TaqMan-MGB probe-based genotyping and Sanger sequencing were performed. Specific primers (ABCD4-Sanger-F and ABCD4-Sanger-R) flanking the SNP site were designed using Primer Premier 5.0 software. Corresponding TaqMan-MGB probes (ABCD4-F, ABCD4-R, ABCD4-FAM and ABCD4-HEX) were developed (Table 1). Based on the traditional TaqMan probe, a minor groove binder (MGB) was incorporated into a non-fluorescent quencher group at the 3′ end. This modification functions through a dual-path mechanism: During PCR overlap, the Taq polymerase’s 5′ → 3′ exonuclease activity cleaves the probe to release fluorescence signals, while the MGB modification strengthens probe-template complex stability through strong binding to the DNA minor groove. All oligonucleotide synthesis and sequencing services were provided by Sangon Biotech Co., Ltd. (Shanghai, China).

#### 2.3.4. Validation of the ABCD4 Gene SNP Locus (Chr7:89393414 C > T) by Sanger Sequencing

To establish a gold-standard genotype dataset for subsequent method comparison, all samples were subjected to Sanger sequencing. Specific primers (ABCD4-Sanger-F and ABCD4-Sanger-R) were designed to amplify the DNA fragment harboring the target SNP. The PCR reaction system (25 μL) consisted of 12.5 μL Green Taq Mix (Vazyme, Nanjing, China, Cat# P131), 1 μL of each primer (10 μM), 1 μL genomic DNA (50–100 ng), and ddH_2_O to adjust the volume. Thermal cycling conditions were as follows: initial denaturation at 95 °C for 3 min; followed by 30 cycles of 95 °C for 15 s, 58 °C for 9 s, and 72 °C for 9 s; with a final extension at 72 °C for 5 min.

PCR products were sent to Sangon Biotech Co., Ltd. (Shanghai, China) for Sanger sequencing using the same primers as in the amplification step. Sequencing results were analyzed using Chromas 2.6.5 software (Technelysium Pty Ltd., Brisbane, Australia). Genotypes were determined by aligning the sequences against the reference genome (NCBI Accession No. 101106333), with manual inspection of peak patterns at the SNP locus (Chr7:89393414).

#### 2.3.5. Construction of TaqMan-MGB Standard Plasmids

The PCR amplification system for standard plasmids (50 µL total volume) consisted of 25 µL of 2× Phanta Max Master Mix (Vazyme, Nanjing, China, Cat# P525-01), 2 µL each of forward and reverse primers (ABCD4-Sanger-F and ABCD4-Sanger-R, final concentration 0.4 μM), 1 µL of template DNA (10 ng), and 19 µL of ddH_2_O. The thermal cycling conditions were as follows: pre-denaturation at 95 °C for 3 min, followed by 30 cycles of 95 °C for 15 s, 57 °C for 15 s, and 72 °C for 3 s, with a final extension at 72 °C for 5 min. PCR products were purified using the EZ-10 column purification kit (Sangon Biotech) and sequenced by Sangon Biotech Co., Ltd. (Shanghai, China). Sequencing results were aligned with the sheep *ABCD4* reference sequence (NCBI Accession No. 101106333) and visualized using Chromas 2.6.5 software (Technelysium Pty Ltd.).

The PCR products identified by Sanger sequencing as homozygous for the C/C or T/T genotype at the *ABCD4* SNP locus (Chr7:89393414) were ligated into the TA/Blunt-Zero Cloning Kit vector (Vazyme, China, Cat# C601-01), incorporating the *ABCD4* gene fragment. The ligation products were gently mixed with 100 µL of Trans1-T1 competent cells, incubated on ice for 30 min, heat shocked at 42 °C for 30 s, then placed on ice for 2 min. Subsequently, 250 µL of LB medium was added, and cells were shaken at 220 rpm at 37 °C for 1 h. After centrifugation, 150 µL of the supernatant was discarded. The remaining suspension was mixed thoroughly and spread evenly onto LB agar plates containing ampicillin. The plates were incubated overnight at 37 °C. Single colonies were picked, resuspended in 10 µL of sterile water, and vortexed. A 20 µL PCR reaction mixture was prepared using the 2×Rapid Taq Master Mix (Vazyme, China, Cat# P222) system, containing 10 µL of 2× Rapid Taq Master Mix, 2 µL of M13 Primer Mix, 2 µL of bacterial suspension, and 6 µL of ddH_2_O. The reaction program was set as follows: pre-denaturation at 95 °C for 3 min; 30 cycles of 95 °C for 15 s, 55 °C for 15 s, and 72 °C for 9 s; final extension at 72 °C for 5 min. PCR products were submitted for sequencing by Sangon Biotech Co., Ltd.

Plasmids confirmed by sequencing to carry homozygous C/C or T/T genotypes at the *ABCD4* locus (Chr7:89393414) were purified using the FastPure Plasmid Mini Kit (Vazyme, China, Cat# DC201-01) according to the manufacturer’s instructions. Plasmid concentrations were quantified via NanoDrop^TM^ 2000 spectrophotometer (Thermo Fisher Scientific), and diluted to a working concentration of 10 ng/µL with nuclease-free water. These plasmid standards served as the gold reference controls for subsequent TaqMan-MGB genotyping validation.

#### 2.3.6. TaqMan-MGB Reaction

TaqMan-MGB genotyping was employed to establish a molecular detection system. The PCR system was built using the ChamQ Geno-SNP Probe Master Mix Kit (Vazyme, Q811-02), with a total volume of 20 µL, including 10 µL of 2× master mix, 1.8 µL of 10 µM forward and reverse primers (ABCD4-F and ABCD4-R), 0.4 µL of 10 µM dual-labeled fluorescent probes (ABCD4-FAM and ABCD4-HEX), 4.6 µL of ddH_2_O, and 1 µL of template DNA (10 ng). Amplification was performed using the Bio-Rad CFX96 real-time PCR detection system with the following thermal profile: 95 °C for 30 s (pre-denaturation), followed by 35 cycles of 95 °C for 10 s and 60 °C for 30 s (annealing and signal acquisition). Each reaction was performed in triplicate. Automatic genotype calling was conducted using the Bio-Rad CFX96 instrument’s accompanying software. In order to validate the reliability of the detection system, blood samples from the 152 Ujimqin sheep were genotyped for molecular marker screening of the multi-vertebrae phenotypes, establishing a TaqMan-MGB genotyping platform suitable for this population.

### 2.4. Data Processing

The association between vertebral phenotypes (T13L6, T13L7, T14L6, T14L7) and genotypes (C/C, C/T, T/T) of the *ABCD4* gene SNP locus (Chr7:89393414 C > T) was assessed using Pearson’s chi-square test. When expected frequencies in any contingency table cell were <5, Fisher’s exact test was applied instead. The strength of association was quantified by calculating odds ratios (OR) with 95% confidence intervals (CI), using T13L6 as the reference phenotype group. Statistical significance was defined at α = 0.05 (two-tailed). All analyses were performed using R software (v4.2.3; R Foundation for Statistical Computing, Vienna, Austria).

## 3. Results

### 3.1. Vertebral Phenotype Detection Using DR Imaging

**Figure 1:**
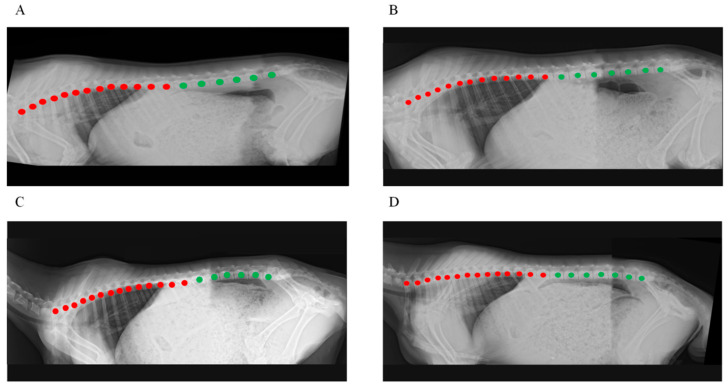
Results of DR imaging detection method for detecting spinal phenotype in Ujimqin sheep: (**A**) T13L6 type Ujimqin sheep; (**B**) T13L7 type multi-vertebra Ujimqin sheep; (**C**) T14L6 type multi-vertebra Ujimqin sheep; (**D**) T14L7 type multi-vertebra Ujimqin sheep; Note: Red dots indicate thoracic vertebrae; green dots indicate lumbar vertebrae.

Most individuals in the Ujimqin sheep population possessed 19 thoracolumbar (the sum of the thoracic and lumbar) vertebrae, comprising 13 thoracic vertebrae and 6 lumbar vertebrae (T13L6) (Figure 1A). A minority of sheep exhibited 20 or 21 thoracolumbar vertebrae, which included three types: 13 thoracic and 7 lumbar vertebrae (T13L7) (Figure 1B), 14 thoracic and 6 lumbar vertebrae (T14L6) (Figure 1C), and 14 thoracic and 7 lumbar vertebrae (T14L7) (Figure 1D). Individuals with 20 or 21 vertebrae were classified as multi-vertebra Ujimqin sheep.

### 3.2. Sequence Analysis of Standard Plasmids

**Figure 2:**
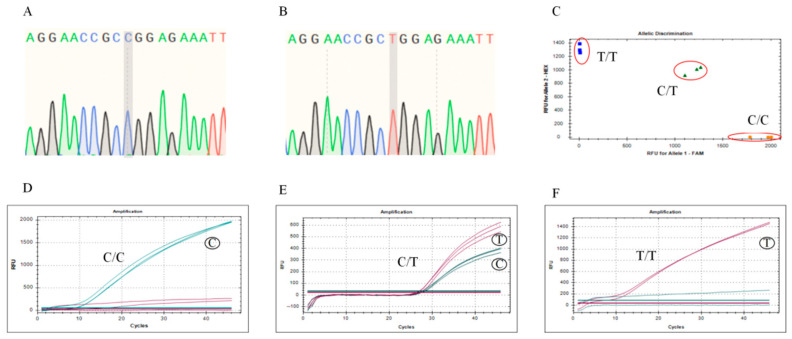
SNP Genotyping of Standard Plasmids: (**A**) Sanger sequencing results of C/C genotype plasmids; (**B**) Sanger sequencing results of T/T genotype plasmids; (**C**) scatter plot confirms clear separation of three genotypes by fluorescence signals, validating assay specificity. The scatter plot of standard plasmids (blue square: SNP (89393414, C > T) locus genotype is “T/T” homozygous; green triangle: SNP (89393414, C > T) locus genotype is “C/T” heterozygous; yellow dots: SNP (89393414, C > T) locus genotype is “C/C” homozygous; red circles: the standard sample); Scatter plot confirms clear separation of three genotypes by fluorescence signals, validating assay specificity. (**D**) Dissolution curve of C/C genotype plasmid; (**E**) dissolution curves of C/C and T/T genotype mixed plasmids; (**F**) dissolution curve of T/T genotype plasmid.

The sequencing strategy successfully identified the correct insertion of homozygous C/C and T/T genotype fragments into the plasmid. The sequencing results are shown in Figure 2A,B. The inserted sequences were in complete agreement with the reference genome, verifying the accuracy of the cloning process. Complementary base-pairing further validated the high fidelity of the inserted fragments, indicating that no mutations were introduced during the construction of the standard plasmids. Genotyping of the standard plasmids was performed using the TaqMan-MGB method. The reaction system was run on the real-time fluorescence quantitative PCR instrument (CFX96) according to preset programs. Analysis via the CFX96 software (CFX Manager Software v3.1)revealed that in the genotyping profile of the standard plasmids, T/T genotype plasmids exhibited a specific Hexachloro-fluorescein (HEX) fluorescence signal (indicated by blue squares), C/C genotype plasmids responded specifically to Fluorescein Amidite (FAM) fluorescence (indicated by yellow dots), and C/T heterozygotes simultaneously displayed both HEX and FAM fluorescence signals (indicated by green triangles), as shown in Figure 2C–F.

### 3.3. TaqMan-MGB Genotyping and Validation

**Figure 3:**
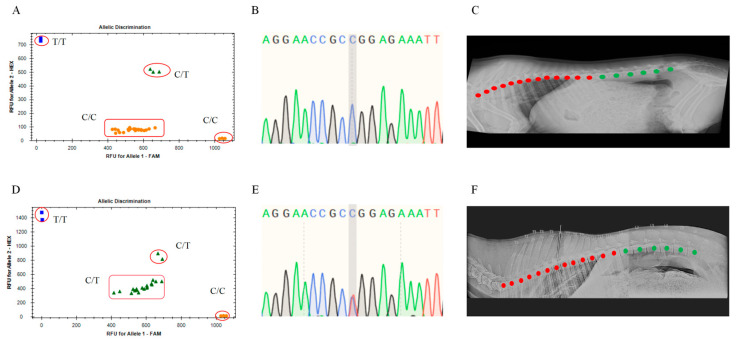
Genetic testing and DR imaging detection results of T13L6 Type Ujimqin Sheep: (**A**) Taqman-MGB genotyping results with specific responses to FAM fluorescence signals; (**B**) Sanger sequencing results of amplification products with specific responses to FAM fluorescence signals; (**C**) DR imaging detection results of Ujimqin sheep with normal vertebral traits; (**D**) Taqman-MGB genotyping results that simultaneously respond to HEX and FAM fluorescence signals; (**E**) Sanger sequencing results of amplification products that simultaneously respond to both HEX and FAM fluorescence signals; (**F**) DR imaging detection results that simultaneously respond to HEX and FAM fluorescence signals. Note: Red dots indicate thoracic vertebrae; green dots indicate lumbar vertebrae.

Genotyping of blood samples from 152 Ujimqin sheep was conducted using the TaqMan-MGB probe assay. The reactions were performed on a real-time fluorescence quantitative PCR system (CFX96) following preset protocols. Analysis with the CFX96 system software revealed that four blood samples exhibited specific FAM fluorescence signals, represented by yellow dots (Figure 3A). Sanger sequencing of the corresponding qPCR amplification products confirmed that these samples carried the C/T genotype (Figure 3B). According to DR phenotyping, the vertebral configuration of these individuals was T13L6 (Figure 3C). Additionally, nine blood samples simultaneously displayed both HEX and FAM fluorescence signals, represented by green triangles (Figure 3D). Sanger sequencing verified these samples also carried the C/T genotype (Figure 3E). DR results similarly indicated a T13L6 vertebral phenotype in these sheep (Figure 3F).

**Figure 4:**
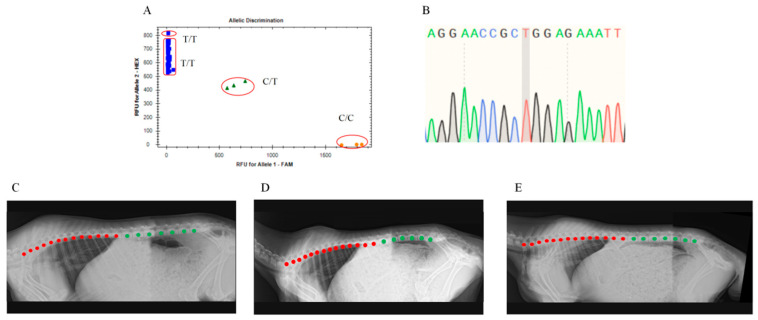
This genetic testing and DR imaging results of T13L7, T14L6, and T14L7 types: (**A**,**B**) Genetic testing results showing HEX fluorescence-specific detection. (**A**) TaqMan-MGB genotyping. (**B**) Sanger sequencing of amplification products. (**C**–**E**) DR imaging detection results of Ujimqin sheep with multi-vertebrae traits. (**C**) T13L7(genotype T/T), (**D**) T14L6(genotype T/T), (**E**) T14L7(genotype T/T). Note: Red dots indicate thoracic vertebrae; green dots indicate lumbar vertebrae.

In addition, the analysis by the CFX96 system software demonstrated that 139 blood samples exhibited specific responses to HEX fluorescence signals, denoted by blue squares (Figure 4A). Sanger sequencing of these real-time quantitative PCR amplification products showed that their genotype was T/T (Figure 4C–E). The corresponding results from DR imaging indicated that these Ujimqin sheep had T13L7, T14L6, and T14L7 types (Figure 4B).

These results verify that the TaqMan-MGB genotyping technology can achieve rapid and accurate identification of the multi-vertebrae traits in Ujimqin sheep, providing reliable technical support for population genetic analysis.

### 3.4. Validation of TaqMan-MGB Genotyping Accuracy and Association Between Genotypes and Phenotypes

In this study, TaqMan-MGB probe assay was used for genotyping 152 Ujimqin sheep. Its reliability was validated against Sanger sequencing (gold standard) and DR imaging results, and the association between genotypes and multi-vertebrae phenotypes was analyzed (Table 2 and Table 3).

As shown in Table 2, the genotyping results of the two methods for the *ABCD4* gene SNP locus (Chr7:89393414 C > T) were completely consistent. The Kappa coefficient for all phenotypes (T13L6, T13L7, T14L6, T14L7) was 1.00 (95% CI: 1.00–1.00), confirming the accuracy and reliability of TaqMan-MGB genotyping.

Chi-square test showed a highly significant association between *ABCD4* genotype distribution and vertebral phenotypes (overall χ^2^ = 76.86, df = 6, *p* < 0.001; Table 3). Specifically: The T/T genotype was highly enriched in multi-vertebrae individuals, accounting for 87.5% (70/80) of T13L7, 97.4% (37/38) of T14L6, and 87.5% (7/8) of T14L7 phenotypes, which was significantly higher than its proportion in the normal T13L6 phenotype (50.0%, 13/26). The CC genotype was only detected in the T13L6 phenotype (23.1%, 6/26) and was absent in multi-vertebrae phenotypes. Odds ratio (OR) analysis showed that individuals with the T/T genotype had 6.50-fold (95%CI: 2.52–16.78, *p* < 0.001), 34.00-fold (95%CI: 4.35–265.20, *p* < 0.001), and 7.00-fold (95%CI: 0.85–57.63, *p* = 0.037) higher risks of exhibiting T13L7, T14L6, and T14L7 phenotypes, respectively, compared with the T13L6 phenotype, with the strongest association observed for the T14L6 phenotype.

## 4. Discussion

Zhou et al. identified an SNP locus in the *ABCD4* gene (Chr7:89393414, C > T) that exhibits a significant mutation in association with the multi-vertebrae trait in Ujimqin sheep [8]. In this study, a TaqMan-MGB genotyping system was successfully developed to target the *ABCD4* SNP (Chr7:89393414, C > T) for the rapid identification of the multi-vertebrae phenotype in Ujimqin sheep. The genotyping results showed 100% concordance with Sanger sequencing—the gold standard for genotyping—and achieved 91.4% accuracy in identifying multi-vertebrae individuals compared with DR imaging. These findings highlight the method’s reliability and its practical utility in molecular breeding.

Notably, minor discrepancies between the two methods may arise due to several factors. On the one hand, the multi-vertebrae trait is regulated by a polygenic network in which the *ABCD4* SNP acts as a major, though not exclusive, determinant. Uncharacterized modifier genes or epistatic interactions may also contribute to phenotypic variation. On the other hand, individual genetic heterogeneity or epigenetic modifications during development could further decouple genotype from phenotype. Despite these potential sources of variation, the overall accuracy remains sufficient for practical breeding applications, enabling efficient screening of individuals with high potential for the multi-vertebrae trait.

While ABCD4 is involved in lysosomal vitamin B12 transport [15,16], and vitamin B12 has been shown to modulate osteogenesis through WNT/Notch signaling pathways [17,18,19,20,21,22], its specific role in vertebral development remains poorly characterized. Given the critical role of somitogenesis in axial patterning, ABCD4-mediated B12 distribution may influence vertebral formation. However, direct mechanistic evidence linking this pathway to polyvertebral traits in sheep is presently limited and requires further functional validation.

The TaqMan-MGB method showed perfect concordance with Sanger sequencing results (Kappa = 1.00), confirming its robustness for targeted SNP genotyping [23]. This high level of agreement is attributed to the MGB modification, which enhances probe–template binding stability and minimizes background fluorescence, thereby improving probe specificity. These findings align with previously reported advantages of MGB-modified probes in detecting single-base mutations [24,25]. Unlike Sanger sequencing, the TaqMan-MGB assay supports high-throughput genotyping and automated analysis via Bio-Rad CFX96 software. Statistical analysis revealed a strong association between the T/T genotype and supernumerary vertebrae phenotypes (T13L7, T14L6, T14L7), with odds ratios (ORs) ranging from 6.50 to 34.00. This finding is consistent with a previous GWAS that identified a significant association (*p* < 10^−6^) between this SNP and vertebral number variation in Ujimqin sheep, further validating the reliability of this SNP by quantifying the strength of the association.

Although both the TaqMan-MGB and DR methods offer high accuracy, DR imaging requires specialized radiographic equipment and advanced expertise in image interpretation, which increases both complexity and cost. In contrast, the TaqMan-MGB approach offers technical advantages by simplifying sample collection and reducing the operational threshold. This technology has been widely applied to SNP detection across various fields. For instance, Tomoko Endo et al. employed it for citrus variety identification [26]. Ma et al. developed a rapid detection system for the Sacbrood virus in China [27]. Huang et al. designed a detection scheme for fungicide resistance in Plasmopara viticola [28], and Yang et al. utilized it for avian influenza virus subtyping [29]. Notably, no such application has yet been reported for vertebral phenotype detection. In this study, we applied the TaqMan-MGB method to detect the multi-vertebrae phenotype in Ujimqin sheep, thereby addressing this research gap.

In summary, the TaqMan-MGB detection system developed in this study demonstrates high-throughput potential and allows for the rapid differentiation between normal and multi-vertebrae phenotypes in Ujimqin sheep. Its standardized workflow and cost-effectiveness provide a robust technical foundation for the development of commercial detection kits targeting the multi-vertebrae trait.

## 5. Conclusions

This study developed a TaqMan-MGB probe-based detection method for the rapid identification of multi-vertebrae phenotypes in Ujimqin sheep and validated the accuracy of the approach. The results demonstrated complete concordance with Sanger sequencing (100%), confirming the method’s high reliability for genotyping. Compared with DR technology, the overall concordance rate was 83.6%, and the detection accuracy for multi-vertebrae individuals (T13L7, T14L6, T14L7) reached 91.4%, highlighting its practical value for trait screening in breeding programs. In conclusion, the TaqMan-MGB detection system established in this study provides a rapid, accurate, and scalable technical tool for the efficient breeding of Ujimqin sheep with multi-vertebrae traits. It not only accelerates the identification of superior individuals during the breeding process but also lays a technical foundation for the future development of commercial detection kits.

## Figures and Tables

**Table 1:** Primer and probe design.

Type	Name	Primer Sequencing	Analysis Type	Amplification Length/bp	Annealing Temperature/°C
Primer	ABCD4-Sanger-F	5′ CAGCCTACCGACTTCAGCAT3′	PCR	319	58
	ABCD4-Sanger-R	5′ TGTGTAATCAACACCCCGCA3′	PCR		
Primer	ABCD4-F	5′ TTCTCTTCTTCAGACCCAGGTTAGA 3′	TaqMan	90	60
	ABCD4-R	5′ TCTGTGATGACCAAGAGGGAAA 3′	TaqMan		
Probe	ABCD4-FAM	5′ 6-FAM-TGCAATTTCTCCGGCG-3′ BHQ1	TaqMan		
	ABCD4-HEX	5′ HEX-TGCAATTTCTCCAGCG-3′ BHQ1	TaqMan		

**Table 2:** Accuracy comparison of TaqMan-MGB detectFion results.

Thoracolumbar Vertebrae Phenotype	Total Detected by DR (*n* = 152)	Genotyping Method	Genotype (*n*, %)			Total (*n*)	Kappa Coefficient (95% CI)
			C/C	C/T	T/T		
T13L6	26	TaqMan-MGB Probe	6 (23.1)	7 (26.9)	13 (50.0)	26	1.00 (1.00–1.00)
		Sanger sequencing	6 (23.1)	7 (26.9)	13 (50.0)	26	
T13L7	80	TaqMan-MGB Probe	0 (0.0)	10 (12.5)	70 (87.5)	80	1.00 (1.00–1.00)
		Sanger sequencing	0 (0.0)	10 (12.5)	70 (87.5)	80	
T14L6	38	TaqMan-MGB Probe	0 (0.0)	1 (2.6)	37 (97.4)	38	1.00 (1.00–1.00)
		Sanger sequencing	0 (0.0)	1 (2.6)	37 (97.4)	38	
T14L7	8	TaqMan-MGB Probe	0 (0.0)	1 (12.5)	7 (87.5)	8	1.00 (1.00–1.00)
		Sanger sequencing	0 (0.0)	1 (12.5)	7 (87.5)	8	

**Table 3:** Association analysis between vertebral phenotypes and *ABCD4* gene SNP genotypes.

Thoracolumbar Vertebrae Phenotype	Total Detected by DR (*n* = 152)	Genotype (*n*, %)			Association Strength		
		C/C	C/T	T/T	χ^2^ Value	*p* Value	OR (95% CI)
T13L6	26	6 (23.1)	7 (26.9)	13 (50.0)	_	_	1.00 (Reference)
T13L7	80	0 (0.0)	10 (12.5)	70 (87.5)	28.62	<0.001	6.50 (2.52–16.78)
T14L6	38	0 (0.0)	1 (2.6)	37 (97.4)	41.35	<0.001	34.00 (4.35–265.20)
T14L7	8	0 (0.0)	1 (12.5)	7 (87.5)	6.89	0.037	7.00 (0.85–57.63)

## Data Availability

The original contributions presented in this study are included in the article. Further inquiries can be directed to the corresponding author(s).

## Abbreviations

The following abbreviations are used in this manuscript:

GWAS	genome-wide association study
MAS	marker-assisted selection
DR	digital radiography
MGB	minor groove binder
LD	linear dichroism
Arg	arginine
Gln	glutamine
FAM	fluorescein amidite
HEX	hexachloro-fluorescein
OR	odds ratios
CI	confidence intervals
